# Prevalence and pattern of focal and potential diffuse myocardial fibrosis in male and female marathon runners using contrast-enhanced cardiac magnetic resonance

**DOI:** 10.1007/s00330-023-09416-3

**Published:** 2023-01-23

**Authors:** Haissam Ragab, Gunnar K. Lund, Lynn Breitsprecher, Martin R. Sinn, Kai Muellerleile, Ersin Cavus, Christian Stehning, Enver Tahir, Stefan Blankenberg, Monica Patten, Axel Pressler, Gerhard Adam, Maxim Avanesov

**Affiliations:** 1 Department of Diagnostic and Interventional Radiology and Nuclear Medicine, University Medical Center Hamburg-Eppendorf, Martinistr. 52, 20246 Hamburg, Germany; 2 Department of General and Interventional Cardiology, University Heart Center, Hamburg, Germany; 3 Philips Research, Hamburg, Germany; 4 Private Center for Sports and Exercise Cardiology, Munich, Germany

**Keywords:** Fibrosis, Hypertrophy, left ventricular, Magnetic resonance imaging, Gadolinium

## Abstract

**Objectives:**

This study analyzed the prevalence and pattern of focal and *potential* diffuse myocardial fibrosis detected by late gadolinium enhancement (LGE) and extracellular volume (ECV) imaging in male and female marathon runners using cardiac magnetic resonance (CMR).

**Methods:**

Seventy-four marathon runners were studied including 55 males (44 ± 8 years) and 19 females (36 ± 7 years) and compared to 36 controls with similar age and sex using contrast-enhanced CMR, exercise testing, and blood samples.

**Results:**

Contrast-enhanced CMR revealed focal myocardial fibrosis in 8 of 74 runners (11%). The majority of runners were male (7 of 8, 88%). LGE was typically non-ischemic in 7 of 8 runners (88%) and ischemic in one runner. ECV was higher in remote myocardium without LGE in male runners (25.5 ± 2.3%) compared to male controls (24.0 ± 3.0%, *p* < 0.05), indicating the potential presence of diffuse myocardial fibrosis. LV mass was higher in LGE + males (86 ± 18 g/m^2^) compared to LGE- males (73 ± 14 g/m^2^, *p* < 0.05). Furthermore, LGE + males had lower weight (69 ± 9 vs 77 ± 9 kg, *p* < 0.05) and shorter best marathon finishing times (3.2 ± 0.3 h) compared to LGE- males (3.6 ± 0.4 h, *p* < 0.05) suggesting higher training load in these runners to accomplish the marathon in a short time.

**Conclusion:**

The high frequency of non-ischemic myocardial fibrosis in LGE + male runners can be related to increased LV mass in these runners. Furthermore, a higher training load could explain the higher LV mass and could be one additional cofactor in the genesis of myocardial fibrosis in marathon runners.

**Key Points:**

*• A high frequency of myocardial fibrosis was found in marathon runners.*

*• Myocardial fibrosis occurred typically in male runners and was typically non-ischemic.*

*• Higher training load could be one cofactor in the genesis of myocardial fibrosis in marathon runners.*

## Introduction

Regular physical activity is generally considered beneficial for the cardiovascular system and is associated with improvement in physiological parameters [1]. Recent reports, however, have drawn attention to possible adverse cardiac effects and increased risk of sudden cardiovascular death events in runners [2–4]. At this current time, there is an incomplete understanding of the relationship between physiological cardiovascular adaption to physical exertion (so-called athletes’ hearts) and potential negative effects due to vigorous exercise [5, 6].

It is recognized that late gadolinium enhancement (LGE) quantified by cardiac magnetic resonance (CMR) imaging occurs in both acute and chronic (scar) infarctions and in an array of other myocardial processes that cause myocardial necrosis, infiltration, or fibrosis [7]. These myocardial processes include myocarditis, hypertrophic cardiomyopathy, amyloidosis, sarcoidosis, and other myocardial conditions [7]. T1 mapping and extracellular volume (ECV) imaging are able to analyze and quantify the increase of extracellular space in various cardiac diseases [8]. It has been shown in patients with left ventricular hypertrophy related to hypertensive heart disease or hypertrophic cardiomyopathy that ECV correlated with a histologically determined degree of myocardial fibrosis [9, 10]. Recent CMR studies in athletes used ECV imaging to study matrix remodeling and the potential presence of diffuse myocardial fibrosis which is not detected by standard LGE [11–14].

Previous CMR studies showed a high prevalence of focal myocardial fibrosis in up to 50% of asymptomatic athletes with still controversial clinical relevance [15–18]. Lifetime amount of exercise, exercise-induced hypertension, and clinically undetected myocarditis were proposed as possible cofactors in the pathogenesis of myocardial scarring [18]. In many cardiac diseases including myocarditis myocardial scar tissue is a risk factor for cardiac arrhythmia and sudden cardiac death [19]. Therefore, a deeper understanding of the causes and clinical relevance of myocardial fibrosis in asymptomatic runners is warranted. Previous CMR studies examined the presence of LGE in marathon runners [5, 20]. However, these studies did not include competitive female runners for comparison, limiting the information about myocardial fibrosis in the growing group of female runners.

The present study analyzed the prevalence and pattern of focal and potential diffuse myocardial fibrosis detected by LGE and ECV in male and female marathon runners using contrast-enhanced CMR.

## Materials and methods

### Ethics and study design

This prospective single-center observational study was approved by the local institutional ethics committee. All subjects gave written informed consent before study participation. The study was performed in accordance with the ethical standards as laid down in the 1964 Declaration of Helsinki and its later amendments.

### Study population

Male and female marathon runners were contacted through advertisements at local running clubs. They were included with a self-reported regular training of at least 10 h per week and a history of at least one completed marathon race. A standard questionnaire was used to assess the weekly training load and lifetime competition history, including completed marathons and finishing times. The best marathon finishing time referred to a marathon distance of 42.195 km defined by World Athletics. Each runner completed at least one marathon with that distance and reported the corresponding finishing time. Controls with similar age and sex were eligible if they exercised less than 3 h per week. Subjects with contraindications for CMR were excluded. All subjects reported no cardiovascular disease and all subjects denied any cardiac or illicit medication intake. All subjects underwent the CMR study before the exercise test, which was performed on the same day. Subjects were instructed to arrive rested with no exercise and no alcohol intake in the preceding 72 h. Any food and caffeine intake was restricted in the preceding 3 h before the CMR and the exercise test was performed 3 h after the CMR.

### Exercise testing and blood samples

Cardiopulmonary exercise testing was performed on the same day after CMR using an eddy current braked cycle ergometer (Ergoselect 100, Ergoline GmbH) to determine maximal oxygen uptake (VO_2max_) and ventilatory threshold. A 12-lead electrocardiogram and heart rate were monitored continuously, and blood pressure was automatically measured every 2 min. The ramp incremental step-exercise test was preceded by a 2-min rest period and unloaded cycling (20 W for 3 min), until a steady state was attained. Depending on the subject´s training history, the ramp was continuously increased by 20 to 40 W/min to bring the participants to the limit of tolerance within 10 to 12 min of exercise.

Blood samples were drawn immediately before the CMR from an antecubital vein in a supine position for 5 min. to obtain hematocrit, creatine kinase, high-sensitive troponin T, and N-terminal pro-brain natriuretic peptide (NT-proBNP).

### CMR protocol

Runners and controls underwent CMR imaging with a 1.5-T Achieva Scanner (Philips Healthcare), using a 5-element cardiac coil and ECG gating. Conventional balanced steady-state free-precession (SSFP) cine imaging in the short axis covering the left ventricle (LV) and right ventricle (RV) was obtained for volumetry and LV mass. A Modified Look-Locker Inversion Recovery (MOLLI) sequence with a 5 s(3 s)3 s scheme on 3 short-axis slices (apical, mid, and basal) before and 15 min after administration of contrast medium was used to perform T1 mapping and ECV quantification. Additionally, after 10 min of a bolus injection of 0.2 mmol/kg gadoterate meglumine (Dotarem, Guerbet), end-diastolic LGE images were acquired with standard phase-sensitive inversion recovery (PSIR) sequences in short axis orientation and in 2-, 3-, and 4-chamber views matching cine images. Details about the scanning protocol were previously reported [21].

### CMR data analysis

Two experienced observers (M.A. experience > 10 years; L.B. experience > 4 years in reading CMRs) independently and blindly analyzed CMR images using cvi42 software (Circle Cardiovascular Imaging Inc.). CMR parameters were normalized to the subject’s calculated body surface area (BSA) and are given as the mean of the two investigators’ measurements. LV and RV volumes and LV mass were measured on short-axis cine images as previously described [22]. Focal myocardial fibrosis was identified on short- and long-axis LGE images and considered present using a threshold method with a cutoff of more than five SDs above normal myocardium [22]. The distribution and pattern of LGE were visually analyzed and reported using a 17-segment model. Native T1 and post-contrast T1 were measured using a single ROI drawn in the septum on a mid-cavity short-axis map and ECV was calculated by the standard formula [23]. Areas of focal LGE were excluded from T1 and ECV quantification to evaluate these parameters unbiased from the presence of LGE [21].

### Statistical analysis

Statistical analysis was performed using GraphPad Prism for MacOS, Version 9.1.1 (GraphPad Software Inc.). All MR imaging data are presented as means of the measurements from two blinded readers. The normality of the data was assessed by Shapiro–Wilk test. Continuous data are presented as mean ± SD, and categorical data are presented as absolute numbers and percentages. Continuous variables were compared between groups using unpaired 2-sided student’s t-tests. For categorical data, the chi-square test or Fisher’s exact test was used as appropriate. Statistical significance was defined as *p* < 0.05.

## Results

### Demographics and CMR characteristics of marathon runners and controls

A total of 74 marathon runners including 19 female runners were enrolled in this study between April 2014 and October 2016. The mean age of the study population was 44 ± 8 years in males and 36 ± 7 years in females, respectively. Another 36 control subjects with a similar distribution of age and sex were included for comparison. Both male and female runners had better performance in exercise tests with higher maximal power and higher VO_2max_ as compared to controls (Table 1). CMR revealed higher LV mass and LV volumes in runners compared to controls. Furthermore, native T1 was lower in male and female runners compared to controls, whereas ECV was higher in male runners at 25.5 ± 2.3% compared to male controls with 24.0 ± 3.0% (*p* < 0.05, Table 1).

**Table 1:** Baseline characteristics of male and female marathon runners and controls

	Male runners (*n* = 55)	Male controls (*n* = 22)	*p values*	Female runners (*n* = 19)	Female controls (*n* = 14)	*p values*
Clinical parameters						
Age, yrs	44 ± 8	40 ± 12	0.0922	36 ± 7	45 ± 12	** < 0.05**
Weight, kg	77 ± 9	80 ± 9	0.1904	59 ± 7	65 ± 10	0.0510
Height, m	1.81 ± 0.07	1.81 ± 0.08	0.9999	1.69 ± 0.07	1.68 ± 0.07	0.6878
BMI, kg/m^2^	23.4 ± 1.9	24.5 ± 2.4	** < 0.05**	20.8 ± 1.7	23.4 ± 3.9	** < 0.05**
BSA, m^2^	1.97 ± 0.14	2.00 ± 0.14	0.3983	1.67 ± 0.12	1.73 ± 0.13	0.1804
Exercise test						
Systolic BP at rest, mmHg	127 ± 14	119 ± 15	** < 0.05**	110 ± 11	125 ± 24	** < 0.05**
Diastolic BP at rest, mmHg	79 ± 10	86 ± 13	** < 0.05**	70 ± 7	81 ± 19	** < 0.05**
Peak systolic BP, mmHg	188 ± 28	175 ± 25	0.0626	170 ± 25	169 ± 20	0.9055
Peak diastolic BP, mmHg	94 ± 20	85 ± 22	0.0881	87 ± 21	100 ± 29	0.1669
Heart rate at rest, bpm	53 ± 8	68 ± 12	** < 0.0001**	52 ± 9	72 ± 11	** < 0.0001**
Peak heart rate, bpm	166 ± 13	164 ± 14	0.5537	173 ± 12	163 ± 14	** < 0.05**
ΔHR rest/peak, bpm	113 ± 13	98 ± 17	** < 0.0001**	122 ± 10	94 ± 13	** < 0.0001**
VO_2max_, mL/kg per min	47 ± 9	38 ± 6	** < 0.0001**	46 ± 10	29 ± 5	** < 0.0001**
Maximal power, W	384 ± 99	236 ± 53	** < 0.0001**	264 ± 43	168 ± 45	** < 0.0001**
Blood parameters						
Hematocrit, %	0.42 ± 0.3	0.44 ± 0.28	** < 0.05**	0.39 ± 0.20	0.41 ± 0.36	0.0547
hs TNT, pg/mL	6 ± 6	5 ± 3	0.4618	5 ± 3	4 ± 1	0.2422
NT-proBNP, pg/mL	39 ± 27	42 ± 28	0.6689	62 ± 44	64 ± 27	0.8823
CMR parameters						
LVEF, %	64 ± 6	64 ± 9	0.8916	67 ± 4	65 ± 5	0.2111
LV mass index, g/m^2^	75 ± 11	67 ± 9	** < 0.001**	59 ± 8	52 ± 7	** < 0.05**
LVEDVi, mL/m^2^	92 ± 13	79 ± 12	** < 0.0001**	84 ± 10	69 ± 9	** < 0.0001**
LVESVi, mL/m^2^	33 ± 8	29 ± 10	0.0694	28 ± 6	24 ± 5	0.0513
LVSVi, mL/m^2^	59 ± 9	49 ± 7	** < 0.0001**	57 ± 7	44 ± 7	** < 0.0001**
RVEF, %	58 ± 7	61 ± 6	0.0815	57 ± 6	63 ± 4	** < 0.01**
RVEDVi, ml/ m^2^	105 ± 15	77 ± 9	** < 0.0001**	95 ± 13	67 ± 10	** < 0.0001**
RVESVi, mL/m^2^	45 ± 11	30 ± 7	** < 0.0001**	40 ± 9	25 ± 5	** < 0.0001**
RVSVi, mL/m^2^	61 ± 9	47 ± 5	** < 0.0001**	54 ± 7	42 ± 6	** < 0.0001**
LGE present, n (%)	7 (13%)	0 (0%)	–	1 (5%)	0 (0%)	–
LGE size, %LV	2.5 ± 1.8	–	–	1.1	–	–
LGE mass, g/m^2^	1.9 ± 1.8	–	–	0.8	–	–
Native T1, ms	982 ± 22	1014 ± 28	** < 0.0001**	999 ± 23	1059 ± 22	** < 0.0001**
Post-contrast T1, ms	508 ± 45	532 ± 73	0.0839	498 ± 21	450 ± 29	** < 0.0001**
ECV, %	25.5 ± 2.3	24.0 ± 3.0	** < 0.05**	27.7 ± 2.1	28.9 ± 3.3	0.2595

Numbers are mean ± SD for continuous and *n* (%) for categorical data. The bold *p* values indicate significant differences between groups

**Abbreviations:** *BMI*, body mass index; *BSA*, body surface area; *BP*, blood pressure; *ECV*, extracellular volume; *HR*, heart rate; *hs TNT*, high-sensitive troponin T; *LV*, left ventricular; *LGE*, late gadolinium enhancement; *LVEF*, left ventricular ejection fraction; *LVEDVi*, left ventricular end-diastolic volume index; *LVESVi*, left ventricular end-systolic volume index; *LVSVi*, left ventricular stroke volume index; *RVEF*, right ventricular ejection fraction; *RVEDVi*, right ventricular end-diastolic volume index; *RVESVi*, right ventricular end-systolic volume index; *RVSVi*, right ventricular stroke volume index; *NT-proBNP*, N-terminal pro-brain natriuretic peptide; *VO*_*2max*_, maximal oxygen uptake

### Prevalence, pattern, and localization of LGE

**Fig. 1:**
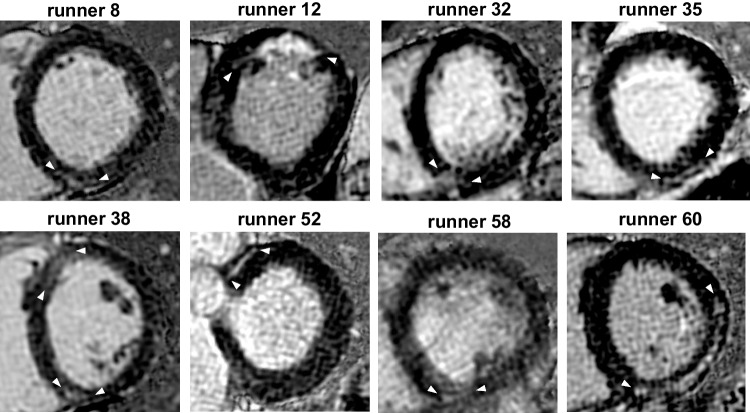
Short axis LGE images of 8 LGE + runners. Non-ischemic LGE was midmyocardial or subepicardial localized in 7 of 8 runners and one runner had subendocardial LGE typical for myocardial infarction (runner 12). Non-ischemic LGE was present in 5 runners at the anterior or posterior RV insertion point (63%, runners 8, 32, 38, 52, 58), in the inferiorlateral wall in 2 runners (25%, athletes 35, 60), and in one runner with ischemic LGE in the anterior wall (12%, runner 12). Runner 38 was the single female runner with LGE, which was located at the anterior and posterior RV insertion points. LGE late gadolinium enhancement, RV right ventricular

**Fig. 2:**
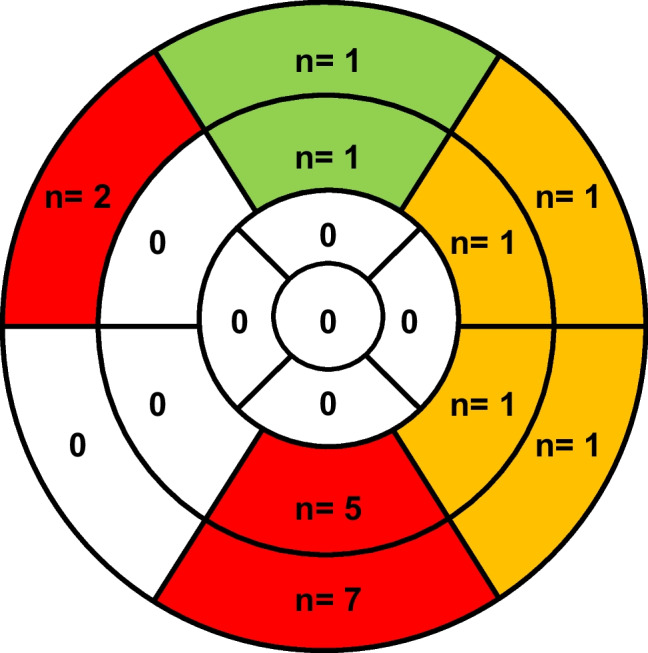
Segmental LGE distribution. The 8 LGE + runners had 20 myocardial segments with LGE. The predominant localization was the anterior or posterior RV insertion point with 12 of 14 inferior segments marked red. Two of these 14 inferior segments were not strictly restricted to the posterior RV insertion point (runner 35 and 60, Fig. 1). The inferolateral and anterolateral wall was involved with 4 segments marked yellow. The anterior wall was involved with 2 segments marked green. LGE late gadolinium enhancement, RV right ventricular

CMR showed focal myocardial fibrosis in 8 of 74 runners (11%) and the majority of these 8 LGE + runners were male (7 of 8, 88%). The prevalence of LGE was 7 in 55 male runners (13%) and one in 19 female runners (5%), however, the LGE difference between both genders was not significant (*p* = 0.14). No LGE was detected in the controls. LGE was midmyocardial or subepicardial localized in 7 of 8 runners (88%). One runner had subendocardial LGE typical for myocardial infarction (runner 12) (12%, Fig. 1). Non-ischemic LGE was present in 5 runners at the anterior or posterior RV insertion point (63%, runners 8, 32, 38, 52, 58), in the inferior-lateral wall in 2 runners (25%, runners 35, 60), and in one runner with ischemic LGE in the anterior wall (12%, runner 12). Runner 38 was the single female runner with LGE, which was located at the anterior and posterior RV insertion points. The mean size of LGE was 2.5 ± 1.8%LV (range 1.0–5.4%LV) and 1.9 ± 1.8 g/m^2^ (range 0.5–5.0 g/m^2^) in male LGE + runners and 2.2%LV and 1.2 g/m^2^ in the one female LGE + runner (Table 1). The 8 LGE + runners had 20 myocardial segments with LGE (mean 2.5 ± 1.5 segments, ranges 1–6). Figure 2 shows segmental LGE distribution in 8 LGE + runners.

### Differences between LGE + and LGE- male marathon runners

LGE + males (*n* = 7) had lower weight (69 ± 9 vs 77 ± 9 kg, *p* < 0.05), lower body surface area (1.85 ± 0.15 vs 1.97 ± 0.14 m^2^, *p* < 0.05), and lower body mass index (21.7 ± 1.9 vs 23.3 ± 1.8 kg/m^2^, *p* < 0.05) compared to LGE- males (Table 2). The heart rate at rest was slightly higher before the exercise test with 60 ± 10 beats per minute compared to LGE- males with 52 ± 7 beats per minute (*p* < 0.05, Table 2). LV mass was higher in LGE + males with 85 ± 17 g/m^2^ compared to LGE- runners with 74 ± 10 g/m^2^ (*p* < 0.05). All other standard CMR parameters including LVEF, LV, and RV volumes were similar between groups. Furthermore, native T1 and ECV values were not different in both groups indicating no incremental potential diffuse myocardial fibrosis in LGE + males. The competition history revealed that the best marathon finishing time was with 3.2 ± 0.3 h shorter in LGE + males compared to LGE- males with 3.6 ± 0.4 h (*p* < 0.05). All other training and competition history parameters were similar in both groups.

**Table 2:** Differences between LGE + and LGE- male runners

	LGE + males (*n* = 7)	LGE- males (*n* = 48)	*p values*
Clinical parameters			
Age, yrs	46 ± 9	44 ± 8	0.5391
Weight, kg	69 ± 9	77 ± 9	** < 0.05**
Height, m	1.78 ± 0.09	1.82 ± 0.07	0.2841
BSA, m^2^	1.85 ± 0.15	1.97 ± 0.14	** < 0.05**
BMI, kg/m^2^	21.7 ± 1.9	23.3 ± 1.8	** < 0.05**
Exercise test			
Systolic BP at rest, mmHg	129 ± 13	126 ± 15	0.6443
Diastolic BP at rest, mmHg	80 ± 11	79 ± 10	0.7726
Peak systolic BP, mmHg	202 ± 29	186 ± 28	0.1515
Peak diastolic BP, mmHg	92 ± 27	95 ± 19	0.7297
Heart rate at rest, bpm	60 ± 10	52 ± 7	** < 0.01**
Peak heart rate, bpm	170 ± 12	165 ± 13	0.3738
ΔHeart rate rest/peak, bpm	110 ± 15	111 ± 21	0.9138
VO_2max_, mL/kg per min	45 ± 13	47 ± 9	0.4731
Maximal power, W	387 ± 109	383 ± 99	0.9239
Blood parameters			
Hematocrit, %	0.43 ± 0.2	0.42 ± 0.3	0.6203
hs TNT, pg/mL	6 ± 3	6 ± 7	0.4377
NT-proBNP, pg/mL	42 ± 18	38 ± 28	0.3040
CMR parameters			
LVEF, %	65 ± 6	63 ± 10	0.7291
LV mass index, g/m^2^	86 ± 18	73 ± 14	** < 0.05**
LVEDVi, mL/m^2^	90 ± 7	90 ± 19	0.6401
LVESVi, mL/m^2^	31 ± 4	33 ± 9	0.6721
LVSVi, mL/m^2^	58 ± 9	58 ± 12	0.9468
RVEF, %	58 ± 6	57 ± 10	0.9276
RVEDVi, mL/m^2^	103 ± 11	104 ± 21	0.8509
RVESVi, mL/m^2^	42 ± 6	44 ± 13	0.7169
RVSVi, mL/m^2^	61 ± 8	60 ± 12	0.8604
Native T1, ms	980 ± 35	963 ± 141	0.7296
Post-contrast T1, ms	502 ± 18	499 ± 87	0.4759
ECV, %	25.4 ± 2.2	25.6 ± 2.2	0.5866
Training + competition history			
Active years, n	12 ± 9	11 ± 6	0.6777
Training, hours per week	12 ± 3	10 ± 4	0.1867
Competitions per year, *n*	13 ± 5	9 ± 6	0.1116
Best marathon time, hours	3.2 ± 0.3	3.6 ± 0.4	** < 0.05**

Numbers are mean ± SD for continuous and n (%) for categorical data. The bold *p *values indicate significant differences between groups

Abbreviations**:** *BMI*, body mass index; *BSA*, body surface area; *BP*, blood pressure; *ECV*, extracellular volume; *HR*, heart rate; *hs TNT*, high-sensitive troponin T; *LV*, left ventricular; *LGE*, late gadolinium enhancement; *LVEF*, left ventricular ejection fraction; *LVEDVi*, left ventricular end-diastolic volume index; *LVESVi*, left ventricular end-systolic volume index; *LVSVi*, left ventricular stroke volume index; *RVEF*, right ventricular ejection fraction; *RVEDVi*, right ventricular end-diastolic volume index; *RVESVi*, right ventricular end-systolic volume index; *RVSVi*, right ventricular stroke volume index; *NT-proBNP*, N-terminal pro-brain natriuretic peptide; *VO*_*2max*_, maximal oxygen uptake

## Discussion

The current study investigated focal and potential diffuse myocardial fibrosis detected by LGE and ECV imaging in male and female marathon runners using contrast-enhanced CMR. The main findings of our study were that eight of 74 runners (11%) had LGE, which was typically non-ischemic with midmyocardial or subepicardial localization (7 of 8 runners, 88%). ECV was slightly higher in male runners compared to controls in remote myocardium without LGE indicating the potential presence of diffuse myocardial fibrosis. LGE + males had higher LV mass compared to LGE- males, indicating a more pronounced adaption of the heart to exercise. LGE + males had lower weight at similar height compared to LGE- males, revealing a more asthenic build in these runners. LGE + males had a shorter best marathon finishing time suggesting a better fitness level for these runners, although exercise testing revealed no difference in performance measured by VO_2max_ and maximal power.

## Prevalence, pattern, and localization of LGE

The prevalence of myocardial fibrosis by LGE was 11% in all marathon runners and 13% in male runners in the current study. The current LGE prevalence is somewhat lower compared to a previous study with 17% in male LGE + triathletes [21]. Furthermore, we found one LGE + female marathon runner compared to none females in triathletes [21]. Our data indicate that male runners are more prone to myocardial fibrosis compared to females, however, the LGE difference between both genders was not significant (*p* = 0.14). One potential explanation is viral myocarditis, which involves males twice as often as females [24]. Despite the fact that all current subjects reported no cardiovascular disease clinically silent or unrecognized myocarditis cannot be excluded per se. In fact, previous work pointed out that elite athletes seem to have an increased risk of viral infection and subsequent myocarditis due to increased exposure to pathogens related to worldwide traveling and international competition or impaired immune system related to continuing training during infections or resuming training early thereafter, strenuous exercise training or competition, and exercising in extreme weather conditions [25]. This assumption for elite athletes could also be true for non-elite athletes.

Besides myocarditis, other factors can be responsible for the preferential occurrence of myocardial fibrosis in males. LGE at the RV insertion points is a frequent finding in patients with pulmonary hypertension [26]. Currently, 63% of our LGE + males had LGE at the anterior or posterior RV insertion point compared to 22% of male triathletes [21]. A previous meta-analysis suggested that besides genetic predisposition and silent myocarditis, pulmonary artery pressure overload and prolonged exercise-induced repetitive microinjury contribute to the development of myocardial fibrosis in runners [18]. Therefore, it is possible that runners develop LGE at the RV insertion points due to exercise-induced pulmonary artery pressure overload resulting in repetitive microinjury. Prior studies pointed out the occurrence of exercise-induced RV cardiomyopathy, which is related to high wall stress of the RV during intense sports, leading to cellular disruption and the release of cardiac enzymes [27, 28]. Our data indicate that myocardial fibrosis at the RV insertion points could be a sequela of the high wall stress of the RV during intense sports. Our CMR data were obtained at rest with no exercise in the preceding 72 h, revealing similar RV volumes in LGE + and LGE- males. Therefore, it is possible that we missed RV dilatation, which typically occurs immediately after exercise [27]. Furthermore, myocardial fibrosis at the RV insertion point may precede significant RV dilation.

The mean size of LGE was smaller in our male runners with 2.5 ± 1.8%LV and 1.9 ± 1.8 g/m^2^ compared to previously studied male LGE + triathletes with 3.5 ± 2.8%LV and 2.9 ± 2.3 g/m^2^ [21]. Our data suggest that runners have less frequent and smaller sizes of LGE compared to triathletes, including iron man participants. This finding could be explained by lower training volume in marathon runners compared to triathletes. Accordingly, a previous study showed that marathon runners have significantly shorter weekly training times and shorter competition times compared to iron man participants [29].

ECV was slightly increased in male runners with 25.5 ± 2.3% compared to controls with 24.0 ± 3.0% in remote myocardium without LGE indicating the potential presence of diffuse myocardial fibrosis. One explanation could be hypertensive heart disease, where there is an expansion of both the myocyte volume resulting from myocyte hypertrophy (intracellular) as well as the interstitial space resulting from fibrosis (extracellular) [30]. However, it has to be stated that the ECV differences were small between the two groups and measurements were within the normal range of ECV measurements. Additionally, our findings about ECV in athletes are different from the data by Swoboda et al. who found decreased ECV values in athletes at 22.7 ± 3.3% compared to controls with 24.3 ± 2.6% [12]. The reasons for these discrepancies in ECV values and the clinical relevance of the observed ECV alterations finding are currently unclear and need further investigation.

One runner had subendocardial LGE typical for myocardial infarction and he received secondary prevention medication. This runner was not aware of having coronary heart disease and he was asymptomatic during exercise and competition. Therefore, we did not recommend invasive or computed tomography coronary angiography for further testing.

## Differences between LGE + and LGE- male marathon runners

LGE + males had lower weight at similar height compared to LGE- males, revealing a more asthenic build in these runners. This finding could be explained by more intensive training in LGE + males, although our standard questionnaire revealed no differences in the weekly training load and the number of active years. However, we found that the best marathon finishing time was shorter in LGE + males compared to LGE- males suggesting a higher fitness level most likely related a more intensive training in these runners. Our standard questionnaire was not designed to document the exact training load before a competition and limited a detailed analysis of the impact of training on the occurrence of LGE. For example, the individual training volume and intensity varies between runners and depends on multiple factors such as motivation, health condition, available time, and so forth. Therefore, a more exact documentation of the individual training absolved by the athlete could be helpful to better understand the training differences in LGE + and LGE- athletes and the impact of training on LGE in athletes in general. The training regimen of athletes is most likely the more important aspect to further study on, since training repeatedly consumes much more time and effort, compared to the “relatively short” marathon distance during one competition.

LV mass was higher in LGE + males compared to LGE- males. A similar finding was previously reported in triathletes [21]. Currently, the exact mechanism for the development of myocardial scarring in athletes is unclear. A previous study suggested that increased systolic blood pressure during exercise and race distances could be cofactors in the origin of myocardial fibrosis [21]. Furthermore, patients with hypertensive heart disease frequently develop non-ischemic myocardial fibrosis [31]. The observed increased LV mass in combination with non-ischemic LGE in our runners could be an expression of hypertensive heart disease, a condition that is so far not related to the athlete´s heart.

## Limitations

We used a standard questionnaire to assess the self-reported training load in our runners. Currently, more precise techniques are available to assess physical activity during training such as quantifying the metabolic equivalent (MET), which was developed to enhance the comparability of results across studies using self-reported physical activity [32]. Future studies are needed to focus on the impact of training on the development of myocardial fibrosis in athletes.

## Conclusions

The current study revealed typically non-ischemic myocardial fibrosis in 7 of 55 (13%) male marathon runners and in one of 19 female runners (5%). Furthermore, we found slightly higher ECV in remote myocardium without LGE in male runners compared to controls demonstrating the potential presence of diffuse myocardial fibrosis possibly related to hypertensive heart disease. No ECV differences were found in female runners and controls. LGE + males had higher LV mass compared to LGE- males, indicating a more pronounced adaption of the heart to exercise. Furthermore, LGE + males had lower weight and shorter best marathon finishing time suggesting a better fitness level and higher training load to accomplish the marathon in a short time. The higher training load can explain the higher LV mass and could be one additional cofactor in the genesis of myocardial fibrosis in athletes. Since the clinical relevance of the observed myocardial fibrosis is currently not clear we did not give any specific recommendation to reduce the individual sports practice in LGE + runners. Further studies are needed to analyze the impact of training load on the occurrence of myocardial fibrosis in athletes and the long-term relevance of myocardial fibrosis on left ventricular function and on the occurrence of relevant cardiac arrhythmia.

## Data Availability

The data of the current study are available upon reasonable request.

## Abbreviations

CMR: Cardiac magnetic resonance
ECV: Extracellular volume
LGE: Late gadolinium enhancement
LGE-: Runner without LGE
LGE +: Runner with LGE
LV: Left ventricle
MET: Metabolic equivalent
MOLLI: Modified Look-Locker Inversion Recovery
NT-proBNP: N-terminal pro-brain natriuretic peptide
RV: Right ventricle
SSFP: Steady-state free-precession
VO_2max_: Maximal oxygen uptake

## Declarations

### Guarantor

The scientific guarantor of this publication is Gunnar K. Lund.

### Statistics and biometry

No complex statistical analysis was performed in this work requiring dedicated statistical support.

### Methodology

• prospective

• observational

• performed at one institution
